# A Facile Synthesis of Fully Protected *meso*-Diaminopimelic Acid (DAP) and Its Application to the Preparation of Lipophilic *N*-Acyl iE-DAP

**DOI:** 10.3390/molecules18011162

**Published:** 2013-01-16

**Authors:** Yukako Saito, Yuichi Yoshimura, Hideaki Wakamatsu, Hiroki Takahata

**Affiliations:** Laboratory of Organic and Pharmaceutical Chemistry, Faculty of Pharmaceutical Sciences, Tohoku Pharmaceutical University, 4-4-1 Komatsushima, Aoba-ku, Sendai 981-8558, Japan; E-Mails: yukako-s@tohoku-pharm.ac.jp (Y.S.); yoshimura@tohoku-pharm.ac.jp (Y.Y.); hiwaka@tohoku-pharm.ac.jp (H.W.)

**Keywords:** protected *meso*-diaminopimelic acid, *N*-acyl iE-DAP, cross metathesis

## Abstract

Synthesis of beneficial protected *meso*-DAP **9** by cross metathesis of the Garner aldehyde-derived vinyl glycine **1b** with protected allyl glycine **2** in the presence of Grubbs second-generation catalyst was performed. Preparation of lipophilic *N*-acyl iE-DAP as potent agonists of NOD 1-mediated immune response from **9** is described.

## 1. Introduction

**Figure 1:**
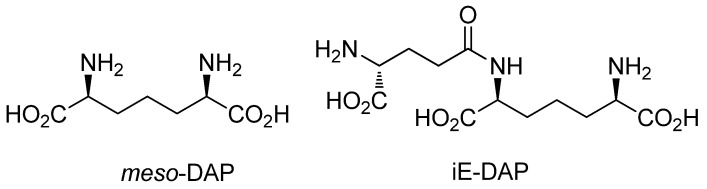
Structures of *meso*-DAP and iE-DAP.

Peptidoglycan (PGN) is an essential component of the cell walls of virtually all bacteria. The function of the PGN is to preserve cell integrity by withstanding the internal osmotic pressure [1,2]. The biosynthesis of PGN is a well-recognized target for antibiotic development [3]. Bacterial cell wall PGN can function as a potent immunostimulator and an adjuvant for antibody production. PGN partial structures are recognized by the intracellular nucleotide-binding oligomerization domain proteins 1 and 2 (NOD1 and NOD2) that mediate host recognition of bacterial molecules [4,5,6]. The recognition core of Nod1 stimulatory molecules is γ-d-glutamyl-*meso*-diaminopimelic acid (iE-DAP) which is a constituent of most Gram-negative and some Gram-positive bacteria. In addition, synthetic, lipophilic, *N*-acyl iE-DAP derivatives have been shown to be potent NOD 1 agonists [7]. Thus, DAP scaffold peptides would be expected to function as NOD 1 agonists. In connection with our interest in the synthesis of DAP [8], we report herein on a new synthesis of orthogonally protected *meso*-DAP and applications to preparing *N*-acyl iE-DAP from protected iE-DAP (Figure 1).

## 2. Results and Discussion

**Scheme 1:**
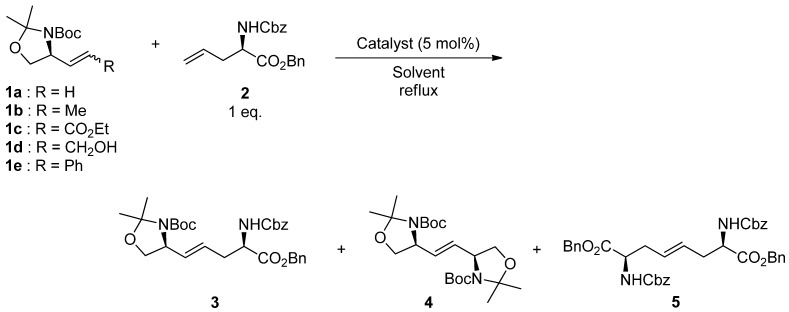
Cross metathesis of Garner aldehyde-derived vinyl glycine equivalents **1** and protected allyl glycine **2**.

Because DAP-containing peptides such as iE-DAP and FK565 [9,10] have significant biological activities and functions, the synthesis of orthogonally protected *meso*-DAP as synthetic intermediates has been a subject of considerable interest by several groups [11,12,13,14,15,16,17,18,19,20,21,22,23,24,25,26]. However, available methods suffer from several disadvantages, including the use of commercially inaccessible starting materials, harsh reaction conditions, multi steps, and lower product yields. We envisaged that orthogonally protected *meso*-DAP could be conveniently prepared by a cross metathesis (CM) between the readily available Garner aldehyde-derived vinyl glycine equivalent **1** and protected allyl glycine **2** (Scheme 1).

**Figure 2:**
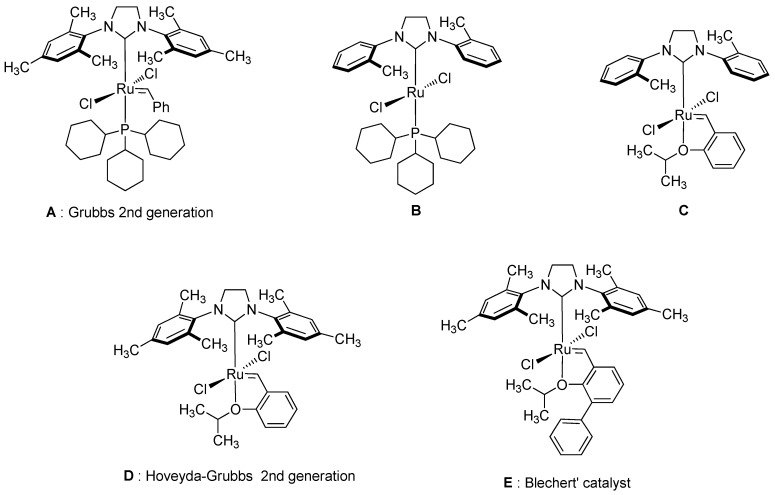
Catalysts for cross metathesis.

A variety of substituted olefins **1a**–**e** as vinyl glycine equivalents were obtained from the Garner aldehyde in high yields according to the corresponding literature reports [27,28,29,30,31]. It is expected that a homocoupling of **1** would scarcely occur due to a bulkiness of *N*-Boc-oxazolidine. First, a coupling began with the CM of **1a** with **2** [32,33]. Treatment of **1a** (5 mmol) with **2** (1 mmol) using the Grubbs second-generation catalyst **A** (5 mol%, Figure 2) in CH_2_Cl_2_ under reflux gave a desired product **3** in 56% together with homo-coupling products traces of **4** and **5** (22%) (entry 1 in Table 1).

Next, the use of a combination of catalysts **B** [34] and **C** [34] gave lower yields (28% and 33%) of **3**, respectively (entries 2 and 3). On the other hand, when toluene was used as the solvent in place of CH_2_Cl_2_, a higher yield (64%) of **3** was obtained (entry 4).

In addition, the use of CM using several substituted olefins **1b**–**e** as vinyl glycine units in toluene was examined and the results are shown in Table 2. The use of **1b** (*E*:*Z* = 1:10) gave the best yield (76%) of **3**. Unfortunately, CM using other derivatives, such as **1c**–**e**, resulted in lower yields (entries 4~6). Furthermore, the CM of **1b** with the Hoveyda-Grubbs 2nd generation **D** [34] and the Blechert **E** [35] catalysts afforded lower yields (56% and 9%), respectively. Accordingly CM in conjunction with a combination of **1b** and **A** as a catalyst resulted in better yields. Additionally, CM using the pure *E* isomer and the *Z* isomer of **1b** resulted in nearly the same yields (75%) (entry 7). Furthermore, the CM in Table 2 produced no the homocoupling product **4** as expected.

With **3** in hand, our interest was focused on the synthesis of orthogonally protected *meso*-DAP. The hydrogenation of **3** in the presence of PtO_2_ as a catalyst gave **6**, which was transformed by hydrolysis of the amino acetal with with *p*-TsOH in aqueous MeOH into the alcohol **7** in 81% yield in two steps. The primary alcohol of **7** was converted into the carboxylic acid **8** by oxidation with TEMPO, which was esterfied, without isolation, with benzyl alcohol using our developed 1-*tert*-butoxy-2-*tert*-butoxycarbonyl-1,2-dihydroisoquinoline (BBDI) [36] to yield the fully protected *meso*-DAP **9** in 94% yield in two-steps.

**Scheme 2:**
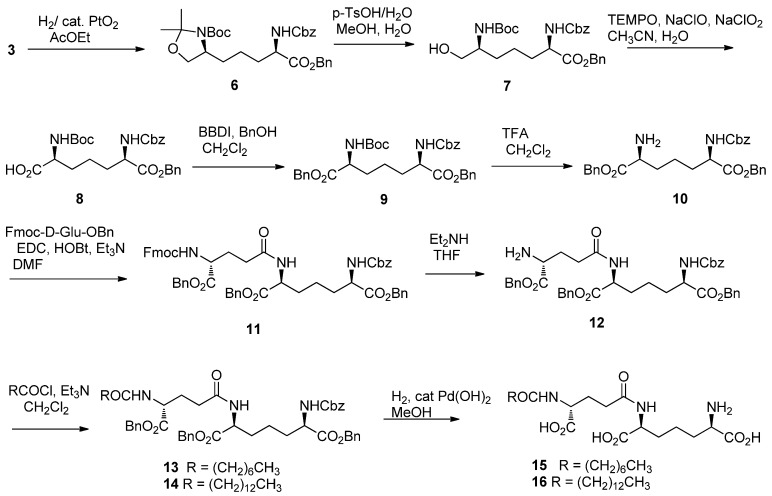
Synthesis of *N*-acyl iE-DAP.

Having the desired **9** in hand, we embarked on the synthesis of *N*-acyl iE-DAP, which is known to function as a strong agonist for the stimulation of NOD 1 [6]. Deprotection of the *tert*-butoxycarbonyl group of **9** by treatment with trifluoroacetic acid followed by condensation of the resulting amine **10** [37] with Fmoc-d-Glu-OBn [38,39] using EDC in the presence of HOBT and triethylamine gave the protected iE-DAP **11** in 54% yield in two steps. Next, **11** was treated with diethylamine to afford the deprotected amine **12**, which was subsequently acylated with capryloyl chloride and myristoyl chloride to produce the corresponding *N*-acyl derivatives **13** and **14** in 68% and 89% yields, respectively. Finally, the deprotection of **13** and **14** with Pd(OH)_2_ as the catalyst under hydrogen gave *N*-capryloyl iE-DAP **15** and *N*-myristoyl iE-DAP **16**, respectively, in quantitative yields (Scheme 2).

## 3. Experimental 

### 3.1. General

Infrared (IR) spectra were recorded on a Perkin-Elmer 1600 series FT-IR spectrometer. Mass spectra (MS) were recorded on a JEOL JMN-DX 303/JMA-DA 5000 spectrometer (Tokyo, Japan). Microanalyses were performed on a Perkin-Elmer CHN 2400 Elemental Analyzer (Tokyo, Japan). Optical rotations were measured with a JASCO DIP-360 or JASCO P-1020 digital polarimeter. Proton nuclear magnetic resonance (^1^H-NMR) spectra were recorded on JEOL JNM-EX 270 (270 MHz) or JEOL JNM-AL 400 (400 MHz) or JNM-LA (600 Mhz) spectrometer (Tokyo, Japan), using tetramethylsilane as an internal standard. The following abbreviations are used: s = singlet, d = doublet, t = triplet, q = quartet, m = multiplet, br = broad. Column chromatography was carried out on Merck Silica gel 60 (230–400 mesh) or KANTO Silica Gel 60N (40–50 μm) for flash chromatography.

### 3.2. Synthesis

*(S)-tert-butyl 2,2-dimethyl-4-vinyloxazolidine-3-carboxylate* (**1a**) [27], *(S)-tert-butyl 2,2-dimethyl-4-(prop-1-en-1-yl)oxazolidine-3-carboxylate* (**1b**) [28], *(S)-tert-butyl 4-(3-ethoxy-3-oxoprop-1-en-1-yl)-2,2-dimethyloxazolidine-3-carboxylate (**1c**)* [29], *(S)-tert-butyl 4-(3-hydroxyprop-1-en-1-yl)-2,2-dimethyloxazolidine-3-carboxylate* (**1d**) [30], *(S)-tert-butyl 2,2-dimethyl-4-styryloxazolidine-3-carboxylate* (**1e**) [31] and *(S)-benzyl 2-(((benzyloxy)carbonyl)amino)pent-4-enoate* (**2**) [32,33] were prepared by the reported procedures.

*(S)-tert-Butyl 4-((R)-5-(benzyloxy)-4-(benzyloxycarbonylamino)-5-oxopent-1-enyl)-2,2-dimethyl-oxazolidine-3-carboxylate* (**3**), (4*S*,4′*S*)-*di-tert-butyl 4,4′-((E)-ethene-1,2-diyl)bis(2,2-dimethyloxazolidine-3-carboxylate)* (**4**), and (2*R*,7*R*,*E*)-*dibenzyl 2,7-bis(((benzyloxy)carbonyl)amino)oct-4-enedioate* (**5**)

To a solution of *N*-Cbz-(*R*)-allylglycine benzyl ester (124 mg, 0.36 mmol) and (*Z*)-(*S*)-*N*-Boc-2,2-dimethyl-4-(1-propenyl)oxazolidine (408 mg, 1.8 mmol) in dry CH_2_Cl_2_ (1.8 mL) was added tricyclohexylphosphine[1,3-bis(2,4,6-trimethylphenyl)-4,5-dihydroimidazol-2-yl-idene][benzylidine]-ruthenium (IV) dichloride (2nd Grubbs catalyst) (15 mg, 0.018 mmol). After the mixture was refluxed for 7 h, the solvent was evaporated. The residue was chromatographed using (*n*-hexane/AcOEt = 2:1) as eluent to yield **4** (17 mg), **3** (109 mg, 56%), and **5** (26 mg, 22%).

**3**: [α]^25^_D_ +9.73° (*c* 1.49, CHCl_3_). IR (neat) cm^−1^: 3329, 2937, 1726, 1697, 1389, 1366, 1255, 1176. ^1^H-NMR (400 MHz, CDCl_3_) δ 1.31–1.56 (15H, m), 2.53–2.60 (2H, m), 3.62 (1H, dd, *J* = 2.17, 8.93 Hz), 3.97 (1H, dd, *J* = 6.04, 8.94 Hz), 4.17 (0.5H, br s), 4.30 (0.5H, br s), 4.44–4.49 (1H, m), 5.06–5.24 (4H, m), 5.35–5.56 (3H, m), 7.34 (10H, s). ^13^C-NMR (100 MHz, CDCl_3_) δ 23.4, 24.8, 26.5, 27.2, 28.1, 28.3, 34.6, 58.6, 66.7, 66.8, 67.0, 67.8, 67.9, 68.1, 79.5, 80.1, 93.4, 93.8, 125.1, 125.6, 128.0, 128.1, 128.1, 128.4, 128.5, 134.1, 135.1, 136.0, 151.7, 155.6, 171.3. EI-MS *m/z* 538 (M^+^). Anal. Calcd for C_30_H_38_N_2_O_7_: C, 66.90; H, 7.11; N, 5.20. Found: C, 66.80; H, 7.31; N, 5.38. 

**4**: [α]^23^_D_ +31.5° (*c* 0.9, CHCl_3_). ^1^H-NMR (400 MHz, CDCl_3_) 1.43–1.83 (30H, m), 3.37 (2H, d, *J* = 8.7 Hz), 4.03–4.05 (2H, m), 4.29–4.44 (2H, m), 5.59 (1H, br s). EI-MS *m/z* 426 (M^+^). HRMS Calcd for C_22_H_38_N_2_O_6_: 426.2730. Found 426.2728.

**5**: m.p. 73–74 °C. [α]^24^_D_ −4.31° (*c* 1.05, CHCl_3_). IR (KBr) cm^−1^:3320, 3064, 3035, 2956, 1741, 1692, 1536, 1346, 1262, 1221, 1052. ^1^H-NMR (400 MHz, CDCl_3_) δ 2.35–2.46 (4H, m), 4.40–4.45 (2H, m), 5.08–5.18 (8H, m), 5.24–5.26 (2H, m), 5.40 (2H, br d, *J* = 7.73 Hz), 7.32 (20H, s). ^13^C-NMR (100 MHz, CDCl_3_) 35.3, 53.4, 66.9, 67.2, 128.1, 128.1, 128.4, 128.5, 128.5 128.6, 135.2, 136.2, 155.7, 171.4. EI-MS *m/z* 650 (M^+^). HRMS Calcd for C_38_H_38_N_2_O_8_: 650.2628. Found 650.2624.

To a solution of *N*-Cbz-(*R*)-allylglycine benzyl ester (139 mg, 0.41 mmol) and (*Z*)-(*S*)-*N*-Boc-2,2-dimethyl-4-(1-propenyl)oxazolidine (497 mg, 2.06 mmol) in dry toluene(3 mL) was added 2nd Grubbs catalyst (18 mg, 0.021 mmol). After the mixture was refluxed for 4 h, the solvent was evaporated. The residue was chromatographed using (*n*-hexane/AcOEt = 3:1) as eluent to yield **3** (168 mg, 76%).

*(2R,6S)-Benzyl 2-(benzyloxycarbonylamino)-6-(tert-butoxycarbonylamino)-7-hydroxyheptanoate* (**6**)

A mixture of **3** (263 mg, 0.49 mmol) in the presence of PtO_2_ (1.8 mg, 0.02 mmol) under hydrogen atomosphere in AcOEt (3.5 mL) was stirred for 14 h at room temperature. After the mixture was filtered through Celite, the filtrate was evaporated to provide (*S*)-*tert*-Butyl 4-((*R*)-5-(benzyloxy)-4-(benzyloxycarbonylamino)-5-oxopentyl)-2,2-dimethyloxazolidine-3-carboxylate (**6**) (257 mg, 97%) as an oil. [α]^25^_D_ +11.18° (*c* 1.2, CHCl_3_). IR (neat) cm^−1^: 3340, 2979, 2938, 1695, 1532, 1456, 1392, 1366, 1257, 1210, 1174. ^1^H-NMR (400 MHz, CDCl_3_) δ 1.26–1.84 (21H, m), 3.61–3.67 (1.5H, m), 3.82 (1H, br s), 4.41 (1H, br s), 5.09 (2H, s), 5.15 (2H, s), 5.42 (0.5H, br d, *J* = 7.25 Hz), 5.60 (0.5H, br d, *J* = 6.76 Hz), 7.33 (10H, s). ^13^C-NMR (100 MHz, CDCl_3_) δ 21.8, 23.1, 24.4, 26.7, 27.5, 28.3, 29.6, 32.0, 32.4, 32.5, 33.1, 53.8, 56.8, 57.0, 66.7, 66.8, 66.9, 67.1, 79.4, 80.1, 93.1, 93.6, 128.0, 128.2, 128.4, 128.5, 135.2, 139.2, 152.3, 155.9, 172.1. EI-MS *m/z* 540 (M^+^).

Seven drops of water were added to a mixture of carboxylic acid (299 mg, 0.55 mmol) and *p*-TsOH·H_2_O (0.07 mmol, 13 mg) in MeOH (5 mL) and then the mixture was stirred for 36 h at room temperature. The whole was evaporated. AcOEt (40 mL) was added to the residue. The mixture was successively washed with *sat*. NaHCO_3_ (10 mL) and brine (10 mL), dried over Na_2_SO_4_, and evaporated. The residue was purified with chromatography using (*n*-hexane/AcOEt = 1:1) as eluent to yield **6** (225 mg, 81%) as solid. M.p. 66–67 °C. [α]^25^_D_ −2.27° (*c* 1.0, CHCl_3_). IR (KBr) cm^−1^: 3353, 2939, 1742, 1694, 1537, 1289, 1247, 1171, 1048. ^1^H-NMR (400 MHz, CDCl_3_) δ 1.31–1.42 (13H, m), 1.54–1.70 (1H, m), 1.80–1.89 (1H, m), 3.45–3.55 (3H, m), 4.39–4.44 (1H, m), 4.83 (1H, br s), 5.09 (2H, s), 5.12–5.21 (2H, m), 5.57 (1H, br s), 7.30–7.36 (10H, m). ^13^C-NMR (100 MHz, CDCl_3_) δ 21.3, 28.3, 30.5, 32.5, 52.0, 53.3, 64.5, 67.0, 67.1, 79.4, 128.0, 128.1, 128.2, 128.4, 128.4, 128.6, 135.2, 136.1, 156.2, 156.2, 172.2. EI-MS *m/z* 500 (M^+^). HRMS Calcd for C_27_H_36_N_2_O_7_: 500.2523. Found 500.2503.

*(2R,6S)-Dibenzyl 2-(benzyloxycarbonylamino)-6-(tert-butoxycarbonylamino)heptanedioate* (**9**)

2,2,6,6-Tetramethylpiperidine 1-oxyl (TEMPO) (7 mg, 0.045 mmol) and 2 M NaClO_2_ (0.43 mL, 0.86 mmol) were added to a solution of **7** (225 mg, 0.45 mmol) in MeCN: sodium phosphate buffer consisted of a 1:1 mixture of 0.67 M NaH_2_PO_4_ and 0.67 M Na_2_HPO_4_ (pH = 6.5 ) (3:2, 6.5 mL) at 40 °C. A diluted solution of commercial bleach (25.5 μL) in H_2_O (485 μL) was then added to the gradually over 0.5 h, and the reaction was stirred at 40 °C for 18 h. The reaction was cooled to room temperature and quenched with sat. *aq.* Na_2_SO_3_ until the mixture became colorless. The solvent was evaporated, the aqueous mixture acidified to pH < 3 with 1 M HCl and extracted with ether (10 mL) five times. Organic layers were washed with brine, dried overNa_2_SO_4_ and evaporated to yield (2*S*,6*R*)-7-(benzyloxy)-6-(((benzyloxy)carbonyl)amino)-2-((*tert*-butoxycarbonyl)amino)-7-oxoheptanoic acid (**8**). Without further purification, a mixture of **8** and BBDI (164 mg, 0.54 mmol) and benzyl alcohol (48 mg, 0.45 mmol) in CH_2_Cl_2_ (2 mL) was stirred for 19 h at room temperature. AcOEt (40 mL) was added to the mixture and the whole was successively washed with 5% HCl (10 mL) and brine (10 mL). The solvent was dried over Na_2_SO_4_ and evaporated. The residue was purified with chromatography using *n*-hexane/AcOEt (3:1) to yield **7** (256 mg, 94%, 2 steps). M.p. 82–83 °C. [α]^24^_D_ −0.28° (*c* 1.4, CHCl_3_). IR (neat) cm^−1^: 3345, 2955, 1741, 1716, 1525, 1367, 1215, 1167. ^1^H-NMR (400 MHz, CDCl_3_) δ 1.22–1.42 (11H, m), 1.53–1.67 (2H, m), 1.75–1.88 (2H, m), 4.26–4.31 (1H, m), 4.34–4.39 (1H, m), 5.02 (1H, br d, *J* = 7.25 Hz), 5.06–5.20 (6H, m), 5.33 (1H, br d, *J* = 7.25 Hz), 7.26–7.34 (15H, m). ^13^C-NMR (100 MHz, CDCl_3_) δ 20.8, 28.2, 31.8, 32.1, 53.0, 53.6, 67.0, 67.1, 79.9, 128.1, 128.1, 128.3, 128.4, 128.5, 128.6, 128.6, 135.2, 135.3, 136.1, 155.4, 155.9, 172.0, 172.3. EI-MS *m/z* 604 (M^+^). Anal. Calcd for C_34_H_40_N_2_O_8_: C, 67.53; H, 6.67; N, 4.63. Found: C, 67.29; H, 6.81; N, 4.43.

*(2S,6R)-Dibenzyl 2-((R)-4-(((9H-fluoren-9-yl)methoxy)carbonylamino)-5-(benzyloxy)-5-oxopentan-amido)-6-(benzyloxycarbonylamino)heptanedioate* (**11**)

TFA (0.6 mL) was added to a mixture of **9** (78 mg, 0.13 mmol) in CH_2_Cl_2_ (0.7 mL) and then the mixture was stirred for 2 h at room temperature. CH_2_Cl_2_ (5 mL) was added to the reaction mixture and the whole neutralized with *sat.* NaHCO_3_. The mixture was extracted with CH_2_Cl_2_, dried over Na_2_SO_4_ and evaporated to yield an amine **10**. Fmoc-d-Glu benzyl ester (59 mg, 0.129 mmol) was added to a solution of the prepared amine **10** in DMF (1.5 mL). Et_3_N (20 μL, 0.142 mmol), HOBt (21 mg, 0.155 mmol), and EDC·HCl (27 mg, 0.142 mmol) were successively added to the mixture at −20 °C. The reaction was stirred for 20 min at the same temperature and then was done at room temperature for 20 h. After addition of AcOEt (100 mL), the mixture was successively washed with 1 N HCl, brine (10 mL × 2), water (10 mL), and 5% NaHCO_3_ (10 mL). The organic layer was dried over Na_2_SO_4_ and evaporated. The residue was purified with chromatography using (*n*-hexane/AcOEt = 1:1) as eluent to yield **11** (70 mg, 57%, 2 steps) as an oil. [α]^23^_D_ −2.0° (*c* 1.6, CHCl_3_). IR (neat) cm^−1^: 3367, 2920, 2851, 1734, 1722, 1662, 1534, 1250, 1211. ^1^H-NMR (400 MHz, CDCl_3_) δ 1.30–1.39 (1H, m), 1.56–1.67 (3H, m), 1.77–1.84 (2H, m), 1.91–2.00 (1H, m), 2.20 (3H, br s), 4.14–4.19 (1H, m), 4.32–4.48 (3H, m), 4.55 (1H, dd, *J* = 7.24, 13.04 Hz), 5.06–5.18 (9H, m), 5.34 (1H, br d, *J* = 7.24 Hz), 5.71(1H, br d, *J* = 7.73 Hz), 6.51 (1H, br d, *J* = 6.76 Hz), 7.29–7.40 (24H, m), 7.58 (2H, br d, *J* = 7.25 Hz), 7.75 (2H, br d, *J* = 7.73 Hz). ^13^C-NMR (100 MHz, CDCl_3_) δ 20.8, 28.6, 31.3, 31.9, 32.0, 47.1, 51.9, 53.3, 53.5, 66.9, 67.0, 67.1, 119.9, 119.9, 125.1, 127.0, 127.7, 128.0, 128.1, 128.2, 128.2, 128.4, 128.5, 128.6, 135.1, 135.2, 135.2, 136.1, 141.2, 141.2, 143.6, 143.8, 156.0, 156.3, 171.8, 171.8, 171.9, 171.9. FAB-MS *m/z* 946 (M^+^+1). HRMS Calcd for C_56_H_55_N_3_O_11_: 946.3870. Found 946.3934.

*(2S,6R)-Dibenzyl 2-((R)-4-heptanecarbonylamino-5-(benzyloxy)-5-oxopentanamido)-6-(benzyloxycarbonylamino)heptanedioate* (**13**)

Et_2_NH (2.5 mL) was gradually added to a solution of **11** (62 mg, 0.066 mmol) in THF (1 mL) with ice cooling over 20 min. The reaction was stirred at room temperature for 3 h and then was evaporated. The residue was purified with chromatography using (CHCl_3_/MeOH = 100:1) as eluent to yield (2*S*,6*R*)-Dibenzyl 2-((*R*)-4-amino-5-(benzyloxy)-5-oxopentanamido)-6-(benzyloxycarbonylamino)heptanedioate (**12**). [α]^24^_D_ −3.1° (*c* 1.0, CHCl_3_). IR (neat) cm^−1^: 3309, 2925, 1739, 1648, 1534, 1215. ^1^H-NMR (400 MHz, CDCl_3_) δ 1.21–1.41 (2H, m), 1.56–1.66 (3H, m), 1.74–1.83 (3H, m), 2.06–2.11 (1H, m), 2.21–2.28 (1H, m), 2.32–2.38 (1H, m), 3.45–3.49 (1H, m), 4.32–4.37 (1H, m), 4.55–4.60 (1H, m), 5.07–5.15 (8H, m), 5.38 (1H, br d, *J* = 7.73 Hz), 6.59 (1H, br d, *J* = 7.73 Hz), 7.30–7.33 (20H, m). ^13^C-NMR (100 MHz, CDCl_3_) δ 20.8, 29.7, 31.6, 31.9, 32.4, 51.7, 53.5, 53.5, 66.7, 67.0, 67.1, 67.1, 128.0, 128.1, 128.2, 128.3, 128.3, 128.4, 128.4, 128.5, 128.6, 128.6, 135.2, 135.5, 136.1, 156.0, 172.0, 172.0, 172.1, 175.4. FAB-MS *m/z* 724 (M^+^+1). HRMS Calcd for C_41_H_45_N_3_O_9_: 724.3189. Found 724.3230.

Et_3_N (9 μL, 0.063 mmol) and *n*-octanoyl chloride (9 μL, 0.053 mmol) were added to a solution of the amine **12** in CH_2_Cl_2_(1 mL) with ice cooling. The reaction was stirred at room temperature for 7 h and then evaporated. After addition of AcOEt (30 mL), the mixture was washed with 10% citric acid (10 mL) and brine (10 mL). The organic solvent was dried over Na_2_SO_4_ and evaporated. The residue was purified with chromatography using (*n*-hexane/AcOEt = 1:1)) as eluent to yield **13** (38 mg, 68%, 2 steps) as an oil. ^1^H-NMR (400 MHz, CDCl_3_) δ 0.87 (3H, t, *J* = 6.76 Hz), 1.19–1.41 (8H, m), 1.54–1.71 (6H, m), 1.75–1.93 (2H, m), 2.16–2.24 (6H, m), 4.33–4.38 (1H, m), 4.55 (1H, dd, *J* = 7.25, 12.57 Hz), 4.73–4.77 (1H, m), 5.09–5.15 (8H, m), 5.50 (1H, br d, *J* = 8.21 Hz), 6.46 (1H, br d, *J* = 7.25 Hz), 7.04 (1H, br d, *J* = 7.73 Hz), 7.29–7.33 (20H, m). FAB-MS *m/z* 850 (M^+^+1). HRMS Calcd for C_49_H_59_N_3_O_10_: 850.4234. Found 850.4278.

*(2S,6R)-Dibenzyl 2-((R)-4-tridecanecarbonylamino-5-(benzyloxy)-5-oxopentanamido)-6-(benzyloxycarbonylamino)heptanedioate* (**14**)

According to the procedure described for a preparation of **13**, a treatment of **11** (71 mg, 0.075 mmol) with Et_2_NH (2 mL) gave **12** (39 mg, 72%), which was converted with Et_3_N (8 μL, 0.058 mmol) and myristoyl chloride (16 μL, 0.058 mmol) into **14** (40 mg, 89%) as a solid. M.p. 90–92 °C. [α]^27^_D_ −4.6° (*c* 1.3, CHCl_3_). ^1^H-NMR (400 MHz, CDCl_3_) δ ^1^H-NMR (400 MHz, CDCl_3_) δ 0.88 (3H, t, *J* = 6.76 Hz), 1.24–1.41 (22H, m), 1.56–1.92 (8H, m), 2.16–2.23 (4H, m), 4.33–4.36 (1H, m), 4.53–4.58 (1H, m), 4.71–4.77 (1H, m), 5.07–5.18 (8H, m), 5.55 (1H, br d, *J* = 8.21 Hz), 6.51 (1H, br d, *J* = 7.73 Hz), 7.06 (1H, br d, *J* = 7.73 Hz), 7.29–7.32 (20H, m). ^13^C-NMR (100 MHz, CDCl_3_) δ 14.1, 21.0, 22.6, 25.6, 29.0, 29.2, 29.3, 29.3, 29.5, 29.6, 29.6, 29.6, 31.1, 31.8, 31.9, 32.2, 36.5, 51.6, 52.0, 53.6, 67.0, 67.0, 67.1, 67.3, 128.0, 128.1, 128.2, 128.4, 128.4, 128.5, 128.6, 128.6, 135.1, 135.2, 135.3, 136.2, 156.0, 172.0, 172.1, 173.9. FAB-MS *m/z* 935 (M^+^+2). Anal. Calcd for C_55_H_71_N_3_O_10_: C, 70.71; H, 7.66; N, 4.50. Found C, 70.73; H, 7.90; N, 4.48.

*(2R,6S)-2-Amino-6-((R)-4-carboxy-4-octanamidobutanamido)heptanedioic acid* (**15**)

A suspension of **13** (34 mg, 0.04 mmol) in the presence Pd(OH)_2_ (25 mg) under hydrogen atomosphere in MeOH (1.5 mL) was stirred for 3 h. The mixture was filtered through Celite. The filtrate was evaporate to yield **15** quantitatively as an oil. [α]^23^_D_ −1.3° (*c* 1.0, MeOH:CHCl_3_ = 1:1). ^1^H-NMR (400 MHz, CDCl_3_) δ 0.90 (3H, t, *J* = 6.28 Hz), 1.31–1.32 (10H, m), 1.52–1.64 (4H, m), 1.73–1.76 (1H, m), 1.85–2.04 (4H, m), 2.14–2.19 (1H, m), 2.23–2.27 (2H, m), 2.35–2.38 (2H, m), 3.68 (br s, 1H), 4.37–4.40 (2H, m). ^13^C-NMR (100 MHz, CDCl_3_) δ 14.4, 22.6, 23.7, 26.9, 28.7, 30.2, 30.3, 31.6, 32.3, 32.9, 33.1, 36.9, 53.5, 53.6, 55.4, 173.8, 175.0, 175.4, 175.6, 176.4. FAB-MS *m/z* 446 (M^+^+1).

*(2R,6S)-2-Amino-6-((R)-4-carboxy-4-tetradecanamidobutanamido)heptanedioic acid* (**16**)

A suspension of **14** (32 mg, 0.034 mmol) in the presence Pd(OH)_2_ (20 mg) under hydrogen atomosphere in MeOH (1.5 mL) was stirred for 3 h. The mixture was filtered through Celite. The filtrate was evaporate to yield **16** quantitatively as an oil. IR (neat) cm^−1^: 3425, 2919, 2850, 1710, 1637, 1538. ^1^H-NMR (400 MHz, CD_3_OD) δ 0.89 (3H, t, *J* = 6.76 Hz), 1.28–1.31 (20H, m), 1.52–1.63 (4H, m), 1.69–1.79 (1H, m), 1.83–2.02 (4H, m), 2.14–2.18 (1H, m), 2.24 (2H, t, *J* = 7.25 Hz), 2.36 (2H, t, *J* = 7.49 Hz), 3.78–3.81 (1H, m), 4.36–4.44 (2H, m). ^13^C-NMR (100 MHz, CD_3_OD) δ 14.4, 22.6, 23.7, 26.9, 28.6, 30.3, 30.5, 30.5, 30.7, 30.8, 30.8, 30.8, 31.3, 32.2, 33.1, 33.1, 36.8, 53.3, 53.4, 54.7, 172.9, 175.0, 175.1, 175.3, 176.5. FAB-MS *m/z* 530 (M^+^+1). HRMS Calcd for C_26_H_47_N_3_O_8_: 530.3447. Found 530.3397.

## 4. Conclusions

In summary, a concise synthesis of *N*-acyl iE-DAP was accomplished starting from the protected *meso*-DAP **9** as a convenient synthon via the use of CM between the Garner aldehyde-derived vinyl glycine **1b** and the protected allyl glycine **2** in nine steps in yields in the range of 22%~29%.

## Figures and Tables

**Table 1:** CM of **1a** with **2**.

Entry	1	Catalyst	Solvent	Time (h)	Yield (%)
1	**1a**	A	CH_2_Cl_2_	7	56
2	**1a**	B	CH_2_Cl_2_	4.5	28
3	**1a**	C	CH_2_Cl_2_	36	33
4	**1a**	A	Toluene	3	64

All reactions were carried out with a ratio (**1a**:**2** = 5:1) under reflux.

**Table 2:** **CM** of Garner aldehyde-derived vinyl glycine equivalents **1b**–**e** with **2**.

Entry	1	Catalyst	Time (h)	Yield (%)
1	**1b**	**A**	4	76
2	**1b**	**D**	3	56
3	**1b**	**E**	4	9
4	**1c**	**A**	4	trace
5	**1d**	**A**	3	9
6	**1e**	**A**	3	10
7	**1b (*E*:*Z* = 10:1)**	**A**	4	75

All reactions were carried out with a ratio (**1**:**2** = 5:1) under reflux in toluene.
